# Ideal chest compression site for cardiopulmonary resuscitation in fontan circulation patients with dextrocardia

**DOI:** 10.1186/s12872-023-03691-0

**Published:** 2024-01-03

**Authors:** Jin Hee Kim, Jae Yun Jung, Sangyun Lee, Soyun Hwang, Joong Wan Park, Eui Jun Lee, Ha Ni Lee, Do Kyun Kim, Young Ho Kwak

**Affiliations:** 1 Department of Emergency Medicine, Seoul National University Hospital, 101 Daehak-ro, Jongno-gu, Seoul, 03080 Republic of Korea; 2 Department of Paediatrics, Seoul National University Children’s Hospital, 101 Daehak-ro, Jongno-gu, Seoul, 03080 Republic of Korea; 3 Department of Paediatrics, Severance Hospital, 50-1, Yonsei-ro, Seodaemun-gu, Seoul, Republic of Korea

**Keywords:** Fontan procedure, Dextrocardia, Cardiopulmonary resuscitation

## Abstract

**Background:**

We aimed to identify the ideal chest compression site for cardiopulmonary resuscitation (CPR) in patients with a single ventricle with dextrocardia corrected by Fontan surgery.

**Methods:**

The most recent stored chest computed tomography images of all patients with a single ventricle who underwent Fontan surgery were retrospectively analysed. We reported that the ideal chest compression site is the largest part of the compressed single ventricle. To identify the ideal chest compression site, we measured the distance from the midline of the sternum to the point of the maximum sagittal area of the single ventricle as a deviation and calculated the area fraction of the compressed structures.

**Results:**

58 patients (67.2% male) were analysed. The mean right deviation from the midline of the sternum to the ideal compression site was similar to the mean sternum width (32.85 ± 15.61 vs. 31.05 ± 6.75 mm). When chest compression was performed at the ideal site, the area fraction of the single ventricle significantly increased by 7%, which was greater than that of conventional compression (0.15 ± 0.10 vs. 0.22 ± 0.11, *P* < 0.05).

**Conclusions:**

When performing CPR on a patient with Fontan circulation with dextrocardia, right-sided chest compression may be better than the conventional location.

**Supplementary Information:**

The online version contains supplementary material available at 10.1186/s12872-023-03691-0.

## Background

Fontan circulation increases the life expectancy of patients born with a single ventricle, which is a structural and functional cardiac anomaly [1, 2]. A previous study on the long-term prognosis of patients with a functional single ventricle showed that the early mortality risk was reduced because of technological advances in the Fontan technique and improvements in perioperative management. Although the late mortality rate decreases over time, the risk of death from late complications, including ventricular failure, protein loss enteropathy, thromboembolism, and sudden death, is still a concern [1]. The absolute risk of sudden cardiac arrest in patients with a single ventricle with Fontan circulation (Fontan patients) is significantly greater than that in the general population (2.1% vs. 0.03 − 0.10% per year) [2, 3]. Therefore, effective cardiopulmonary resuscitation (CPR) is more important for patients with Fontan circulation.

Effective CPR includes an adequate compression rate and depth and minimal pauses during compressions, thus allowing the chest wall to fully recoil after each compression and avoiding excessive ventilation. Additionally, the position of the hand or fingers is important to improving compression effectiveness [4]. According to the 2020 CPR guidelines, the proper chest compression point is the lower half of the sternum in children and adults, except for infants [5, 6]. However, the chest compression point recommended in the current guidelines was the same for all cardiac arrest patients regardless of cardiac anomaly, including Fontan circulation patients with dextrocardia [7, 8]. In a recent study based on computed tomography(CT) analysis, researchers reported that the optimal compression point in patients with Fontan circulation who underwent CPR was approximately 5–25% of the lower sternum, which was lower than the suggested location in the current guidelines; however, patients with dextrocardia, defined as cardiac positioning with a rightward base-apex axis, were not considered in the study [9–11]. Additionally, there have been no relevant studies on compression points in patients with dextrocardia.

The objective of this study was to investigate the ideal chest compression site for CPR in Fontan circulation patients with dextrocardia and to compare the effects of CPR at the proposed location with those of CPR at the site recommended in current guidelines.

## Methods

### Study setting and data source

This was a retrospective cross-sectional observational study of Fontan circulation patients identified using a registry in a tertiary academic hospital in Korea.

### Study population and study design

**Fig. 1:**
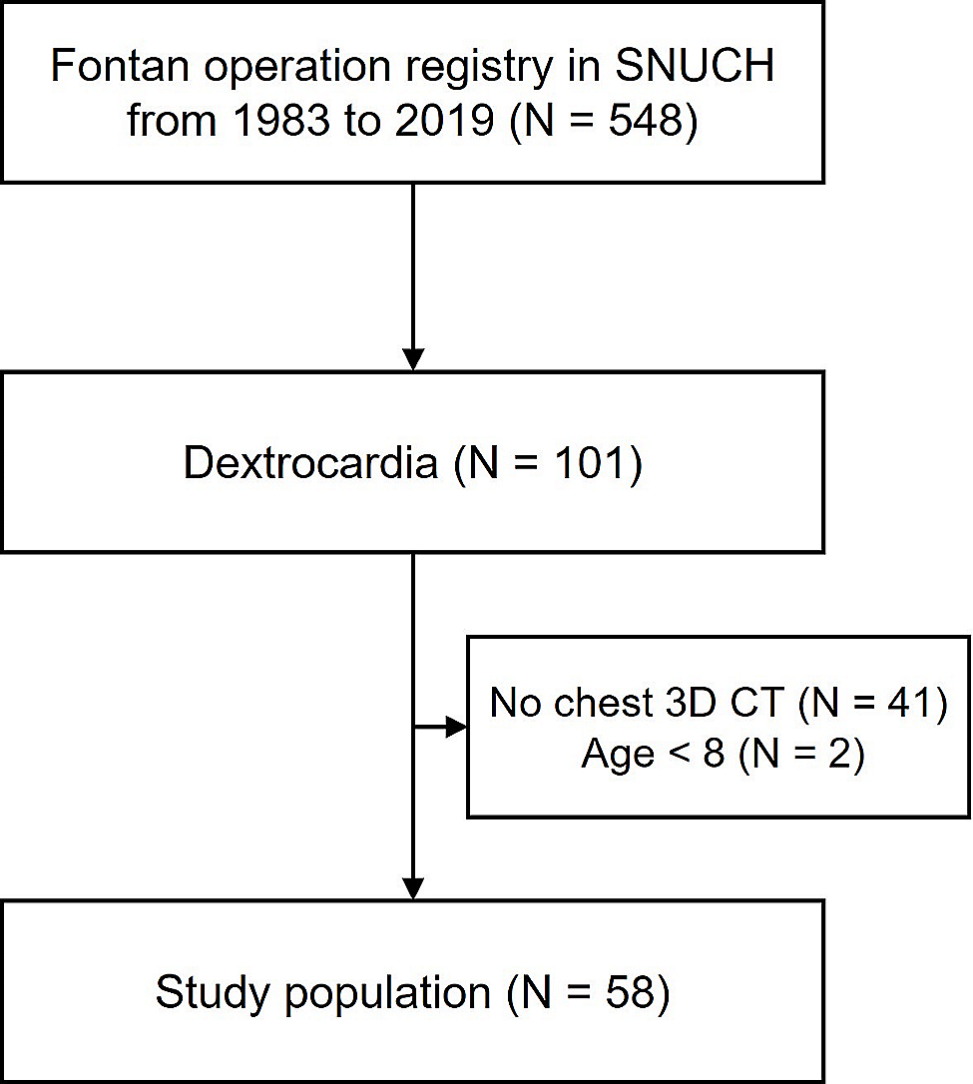
Patient flow SNUCH, Seoul National University Children’s Hospital; 3D CT, Three-dimensional computed tomography

According to domestic statistics in 2019, 1765 Fontan surgeries were performed in Korea, 548 of which were performed at Seoul National University Children Hospital (SNUCH) between January 1983 and December 2019. Among the surgeries that were performed, we retrospectively analysed 101 patients diagnosed with Fontan circulation with dextrocardia at SNUCH during the same period. The inclusion criteria were as follows: patients with dextrocardia among those who underwent Fontan surgery and who underwent contrast-enhanced chest CT. We excluded 41 patients for whom the cardiac cross-sectional area could not be measured without contrast-enhanced chest CT, as the absence of contrast agent prevented the differentiation between various ventricular and atrial structures within the heart. In addition, two patients were excluded because the quality of the CT scans was insufficient to differentiate cardiac structures. Finally, we retrospectively analysed 58 patients (Fig. 1).

### Variables

Demographic data, including date of birth, sex, height, weight, date of operation, date of CT evaluation, initial palliative surgical procedure, and follow-up information, such as survival, death and lost to follow-up, were collected from electronic medical records. We also examined the anatomical variation and dominant diagnosis in each patient’s heart by reviewing echocardiographic and medical records. Anatomical variation included ventricular dominance, laterality of the aortic arch, structural defects such as valve and septal defects, and situs classification. In this study, age was defined as the date of the most recent CT scan and was calculated as the difference between the date of the most recent CT scan and the date of birth. Patient height and weight were obtained from the most recent CT consent form.

### CT scan and outcome measurements

All CT images were exported in Digital Imaging and Communications in Medicine (DICOM) format by an INFINITT Picture Archiving and Communications System (PACS) (version 5.0.0.21, INFINITT Healthcare Co., Ltd.), followed by three-dimensional (3D, Siemens) reconstruction. The region of interest was drawn manually on each slice of the axial and sagittal CT scans. We finally confirmed dextrocardia and the anatomical relationship between the aorta and the heart (e.g., right aortic arch or left aortic arch) using CT images. We defined the ideal chest compression site as the largest area of the compressed single ventricle.

**Fig. 2:**
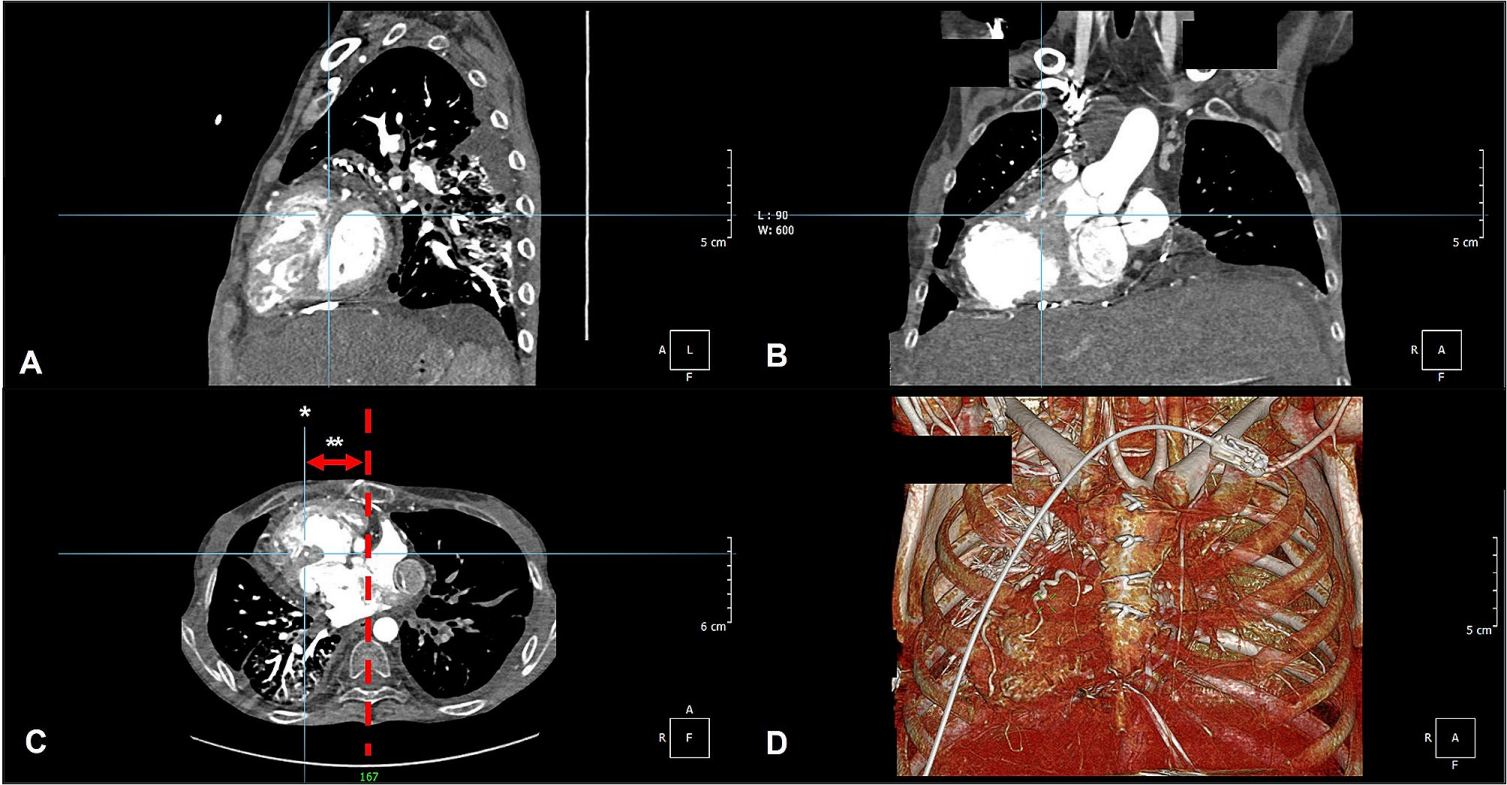
DORV with dextrocardia in a female aged 33 years. Definition of the site and deviation: The site of the single ventricle maximum area is shown in Fig. 2A, the sagittal section corresponds to the site (*), and axial section in Fig. 2C. In Fig. 2C, the midline of the sternum is indicated by a red dotted line and the deviation is indicated by two asterisks (**). We also confirmed the anatomy by referring to the site in the coronal view (Fig. 2B) and the 3D reconstruction view (Fig. 2D).DORV, Double outlet right ventricle

**Fig. 3:**
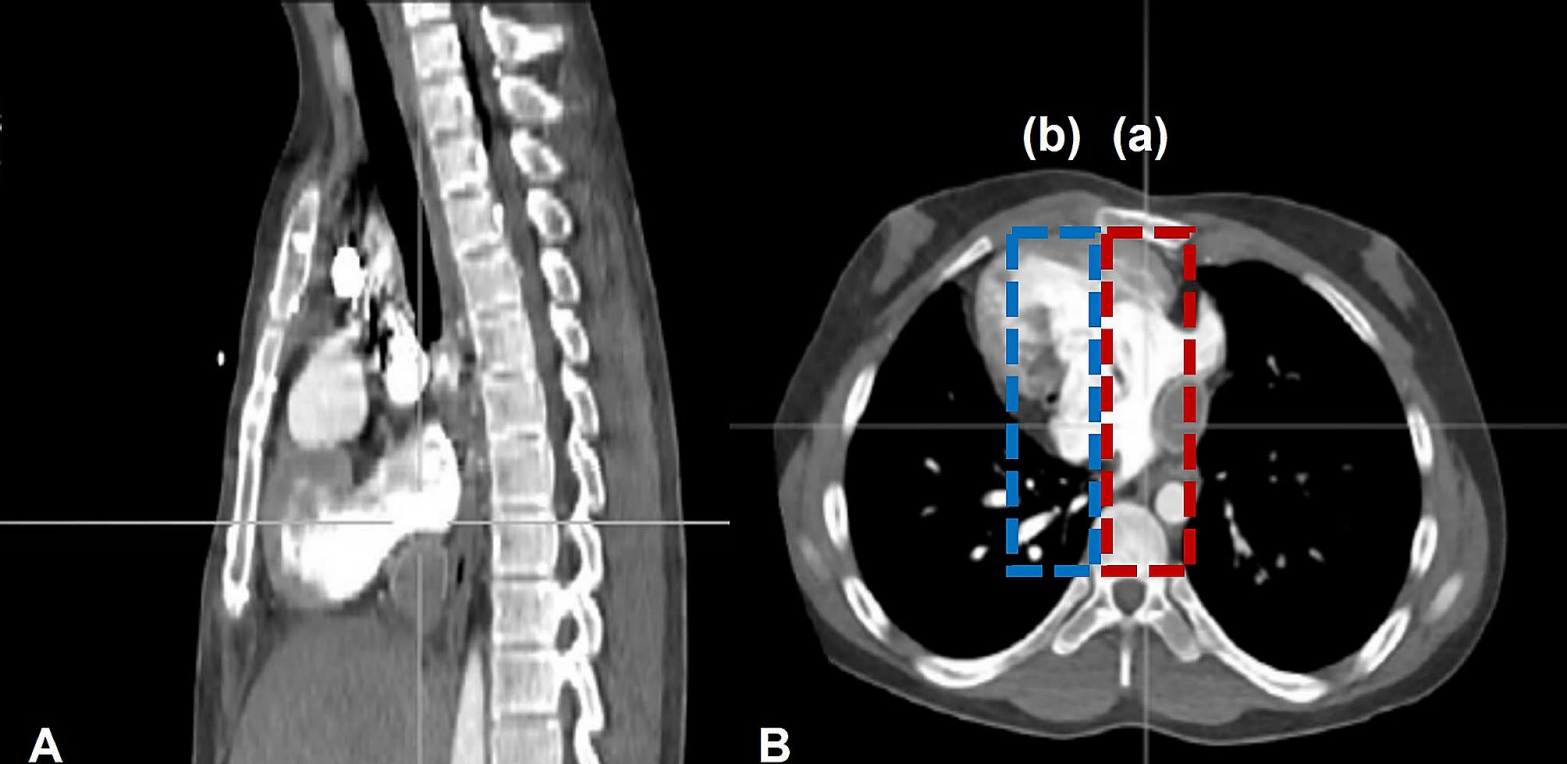
DORV with dextrocardia in a male aged 13 years. Measurement of areas on chest CT images. After confirming the position of the lower quarter of the sternum in the sagittal section (**A**), the area of ​​each heart structure was measured in the corresponding axial section (**B**). In Fig. 3B, (**a**) the predicted compressed area of each structure of the heart behind the sternum (the red dashed line) at the same level and (**b**) the predicted compressed area of each structure of the heart after moving the width of the sternum at the right costosternal junction (the blue dashed line) are shownDORV, Double outlet right ventricle

To find the ideal chest compression site, we found the point where the area of ​​a single ventricle was the largest on the sagittal section (Fig. 2A) and measured the distance from the midline of the sternum (red dotted line in Fig. 2C) to the point (asterisk (*) in Fig. 2C) corresponding to the axial section. We defined this distance as the deviation (two asterisks (**)) in Fig. 2C. In some cases, because there was a point where the area of ​​a single ventricle was at its maximum to the left of the midline of the sternum, we represented these cases as negative numbers. Then, we measured the axial sternum width in each patient at the same location and compared the mean deviation and the mean sternal width. In Fig. 2, we present the PACS screen used in our hospital for reference and confirm the anatomy by referring to points in the coronal section (Fig. 2B) and 3D reconstruction view (Fig. 2D).

Next, to measure the compressed area of each cardiac structure in the total heart area (THA) when compressing conventional and ideal sites, we measured the area of ​​the single ventricle (SVA), the atria (AA), and the great vessels (GVA), including the aorta and the Fontan conduit. We separated each area according to the heart valves. Based on a previous study on the optimal compression position for single ventricle patients, we measured all areas at the lower quarter of the sternum (25%) on sagittal sections (Fig. 3A) [9]. We defined the THA at that location as the sum of the areas of the SVA, the AA, and the GVA. Additionally, we measured the compressed area of ​​the structures directly behind the sternum during CPR (Fig. 3B(a)): the single ventricle area compressed (SVAc), the atria area compressed (AAc), and the great vessels area compressed (GVAc). Since the mean deviation and the mean of the sternum width are similar, we decided to define the shift in sternum width from the right costosternal junction as the ideal site of chest compression and then measured the area predicted to be compressed (Fig. 3B(b)): the single ventricle area compressed under the ideal chest compression site (SVAci), the atria area (AAci), and the great vessels area (GVAci).

Finally, the area fraction of each compressed heart structure was calculated by dividing the area of ​​each compressed heart structure measured with THA at the conventional location of compression and the ideal compression site. We present the following findings in this study: SVAcF, AAcF, and GVAcF.

We also assessed the visibility of the liver in the same cross-section in each patient to check for possible liver damage during chest compressions.

### Statistics

Continuous variables are expressed as the mean ± standard deviation, and categorical variables are expressed as the frequency and percentage of total cases. Additionally, to compare the area fraction of each point within the same subject, we used a paired t-test to determine the differences in continuous variables between the groups. All data were analysed by using R software (version 4.1.2). A two-tailed, *P* value < 0.05 was considered to indicate statistical significance.

### Human ethical approval and informed consent

The study complied with the Declaration of Helsinki, and its protocol was approved by the Seoul National University Hospital Institutional Review Board (No. 2201-044-1288) with a waiver of informed consent.

## Results

### Demographic findings

A total of 58 subjects were included in this study. Among the participants, 39 (67.2%) were male. The mean age was 22.23 ± 8.84 years, the mean height was 160.08 ± 17.31 cm, and the mean weight was 53.14 ± 16.49 kg. We investigated the data of 51 patients who visited our institution for follow-up, and four patients were not followed up due to the distance between home and our institution. In addition, three patients died immediately after surgery. All patients underwent the Fontan operation. Twenty-six bidirectional cavopulmonary shunt (BCPS) operations, 9 Blalock–Taussig shunt (BT shunt) operations and 2 Kawashima operations were performed before the Fontan operation. The anatomical variationand dominant diagnoses of the patients who underwent the Fontan operation are described in Table 1.

**Table 1:** Basic characteristics of the study population

Characteristics	
Number of patients, n (%)	58 (100)
Sex, n (%)	
Male	39 (67.2)
Female	19 (32.8)
Age at recent chest CT (mean ± SD)	22.23 ± 8.84
Height at recent chest CT (mean ± SD)	160.08 ± 17.31
Body weight at recent chest CT (mean ± SD)	53.14 ± 16.49
Follow up, n (%)	
Survival	51 (87.9)
Death	3 (5.2)
Lost to follow-up	4 (6.9)
Operation, n (%)	
Fontan operation	58 (100.0)
BCPS operation	26 (44.8)
BT shunt operation	10 (17.2)
Kawashima operation	2 (3.4)
Dominant ventricle, n (%)	
Right	27 (46.6)
Left	3 (5.2)
Unrecorded	28 (48.3)
Aorta, n (%)	
Right aortic arch	15 (25.9)
Left aortic arch	43 (74.1)
Complete atrioventricular valve, n (%)	9 (15.5)
Septal defects, n (%)	
Ventricular septal defect	14 (24.1)
Complete atrioventricular septal defect	16 (27.6)
Pulmonary valve, n(%)	
Pulmonary stenosis	33 (56.9)
Pulmonary atresia	11 (19.0)
Visceral situs, n(%)	
Solitus	42 (72.4)
Inversus	9 (15.5)
Ambiguous(heterotaxy)	7 (12.1)
Dominant diagnosis, n(%)	
Double-outlet right ventricle	17 (29.3)
Complete atrioventricular septal defect	16 (27.6)
Transposition of great arteries	13 (22.4)
Double-inlet left ventricle with double-outlet right ventricle	4 (6.9)
Double-inlet left ventricle	2 (3.4)
Double-inlet right ventricle	2 (3.4)
Pulmonary atresia	2 (3.4)
Double-inlet right ventricle with double-outlet right ventricle	1 (1.7)
Tricuspid atresia	1 (1.7)

SD, standard deviation

### Main analysis

The mean right deviation from the midline of the sternum to the ideal compression site was 32.85 ± 15.61 mm (similar to the mean sternum width, 31.05 ± 6.75 mm).

The mean SVA of ​​all the subjects was 4030.65 ± 1174.90 mm^2^, the mean AA was 2129.39 ± 902.64 mm^2^, and the mean GVA was 875.16 ± 528.72 mm^2^. The mean THA, which was the mean of the total sum of the areas, was 6952.36 ± 1707.61 mm^2^. The mean area of ​​each compressed heart structure at the conventional location of compression was as follows: SVAc, 988.34 ± 703.15 mm^2^; AAc, 913.43 ± 496.89 mm^2^; and GVAc, 335.64 ± 308.26 mm^2^.

When the chest compressions were performed by moving to the right by the sternum width, the mean area of each compressed cardiac structure was as follows: SVAci, 1476.81 ± 608.40 mm^2^; AAci, 563.39 ± 559.92 mm^2^; and GVAci, 563.39 ± 559.92 mm^2^ (Table 2).

**Table 2:** The average area of cardiac structures and the compressed cardiac structures

	Area	Dextrocardia (n = 58)	Age < 18 (n = 22)	18 ≤ Age (n = 36)
Cardiac chamber (mm^2^, mean ± SD)	THA	6952.36 ± 1707.61	6150.5 ± 1477.34	7442.38 ± 1669.78
	SVA	4030.65 ± 1174.90	3776.55 ± 1379.9	4190.37 ± 1014.64
	AA	2129.39 ± 902.64	1794.39 ± 1025.78	2334.12 ± 762.83
	GVA	875.16 ± 528.72	579.65 ± 253.35	1055.75 ± 572.99
Classic location of compression (mm^2^, mean ± SD)	THAc	2237.37 ± 862.33	1801.45 ± 718.14	2503.76 ± 842.11
	SVAc	988.34 ± 703.15	811.23 ± 666.53	1096.58 ± 711.95
	AAc	913.43 ± 496.89	788.41 ± 489.42	989.83 ± 492.49
	GVAc	335.64 ± 308.26	201.88 ± 214.96	417.38 ± 330.02
Ideal site of compression (mm^2^, mean ± SD)	THAci	2224.87 ± 610.61	2030.13 ± 667.33	2343.88 ± 549.18
	SVAci	1476.81 ± 608.40	1383.05 ± 777.70	1534.11 ± 480.65
	AAci	563.39 ± 559.92	242.52 ± 316.58	567.24 ± 598.84
	GVAci	184.66 ± 279.95	89.98 ± 174.79	242.52 ± 316.58

SD, standard deviation; THA, total heart area; SVA, area of the single ventricle; AA, area of the atria; GVA, area of the great vessels; THAc, total heart area compressed; SVAc, single ventricle area compressed; AAc, the atria area compressed; GVAc, the great vessels area compressed; THAci, total heart area compressed under ideal site chest compression; SVAci, single ventricle area compressed under ideal site chest compression; AAci, atria area compressed under ideal site chest compression; GVAci, great vessels area compressed under ideal site chest compression

A comparison of the area fraction of the compressed cardiac structures at each site is shown in Table 3. At our ideal site of compression, the area fraction of the compressed single ventricle increased by 7%, which was a statistically significant difference (SVAcF, 0.15 ± 0.10 vs. SVAcFr, 0.22 ± 0.11; *P* < 0.05).

**Table 3:** Comparison of the area fraction of compressed cardiac structures at each location

	Conventional site of compression	Ideal site of compression	*P* value*
SVAcF (mean ± SD)	0.15 ± 0.10	0.22 ± 0.11	< 0.05
AAcF (mean ± SD)	0.13 ± 0.07	0.08 ± 0.08	< 0.05
GVAcF (mean ± SD)	0.05 ± 0.04	0.03 ± 0.05	< 0.05

*Statistical significance was tested by paired t-tests between groups

SVAcF, the area fraction of single ventricle area compressed; AAcF, the area fraction of atria area compressed; GVAcF, the area fraction of great vessels area compressed

The liver was not visible in the cross-sectional area in any of the patients.

## Discussion

According to our study, the mean right deviation from the midline of the sternum at the ideal compression site was similar to the mean sternum width (32.85 mm vs. 31.05 mm). Note that, here, there were three left dominant ventricle cases, with ‘deviation’ values of 4.9 mm, 24.1 mm, and 26.1 mm; all positive values are shown. There was only one case with a negative “deviation” value of -4.9 mm in the case of the unrecorded dominant ventricle. This finding confirmed that the “deviation” itself was not caused by the dominance of the ventricles in the 58 patients.

For Fontan circulation patients with dextrocardia, the ideal chest compression site can be identified by moving to the right by the width of the sternum, which is better for CPR than the conventional chest compression position because it is helpful to increase the compressed single ventricle area by more than 7%.

According to the cardiac pump theory, an increase in the cross-sectional area of ​​the ventricle during compression leads to an increase in cardiac output [12]. An increase in cardiac output will help lead to the return of spontaneous circulation in cardiac arrest patients. In the 2020 Pediatric Advanced Life Supports (PALS) guideline, researchers emphasized the need for a single ventricular preload that relies on passive flow across the pulmonary vascular bed during the resuscitation of single ventricle patients who underwent Fontan surgery [5, 6]. As shown in Table 3, the area fraction of the great vessel area, including the Fontan tract, was also significantly lower when the point of chest compression was moved to the right by the width of the sternum. The Fontan tract is less compressed, so the preload can be secured during CPR [13]. Therefore, the results of our study can support the background theory of the 2020 PALS guidelines. In addition, this approach may be more helpful for Fontan circulation patients with dextrocardia.

In previous studies, researchers reported that although dextrocardia is rare in the general population, dextrocardia affects up to 25% of patients with univentricular anatomy. In our study, the incidence of dextrocardia in Fontan patients from 1983 to 2019 was approximately 18% (101/548). The mortality of Fontan circulation patients has decreased due to the recent development of the Fontan procedure and the development of perioperative treatment and management. However, according to a single institution study of patients with single-ventricle physiology and dextrocardia reported in 2014 by Chin L et al., only 56% of patients aged 15 years or younger with this condition survived, and the surgical outcome was poor [10]. Therefore, effective CPR for these patients is still important in emergency medicine. Recently, precision medicine has become the focus of increased attention. Moreover, even if resuscitation begins according to general guidelines, precision resuscitation should be considered. In precision resuscitation, the anatomy and physiology of the patient are considered and an individual approach relating to the patient’s response to resuscitation is used.

### Limitations

This study has several limitations. First, in this study, we retrospectively reviewed the medical records of patients treated at a single institution. However, patients reviewed in the study accounted for approximately 30% of patients who underwent Fontan surgery at the our hospital in Korea in 2019. Although few Fontan operations have been performed, the number of patients investigated in this study was not small. In addition, the strength of our study is significant because there are no previous studies in which researchers attempted to identify the ideal location of compression for CPR in Fontan circulation patients with dextrocardia.

Second, we measured the site at which the area of ​​a single ventricle was the largest, but we could not present this value as a ratio to the total thorax because the entire thorax, including the chest wall, was not visible on any of the analysed CT scans in this study. The CT protocol used to obtain the data was limited, because the purpose of CT was to identify the anatomical location of blood vessels or thrombosis in the Fontan tract in these patients. However, using this contrast CT protocol, we were able to better distinguish heart structures. Instead, when we calculated the body mass index using height and weight at the time of CT in all the patients, the points representing the maximum area of the single ventricle of each patient, regardless of age, were located on the right side and approximately 20–40 mm from the midpoint of the sternum. These findings support our argument that the efficacy of moving to the right by the sternum is similar width regardless of age.

Third, real-world applications may differ because we did not measure the area of the compressed ventricle in real time during CPR. To assess the dynamic aspects of CPR, such as real-time compressibility or potential complications of compressions, we suggest using a high-fidelity CPR manikin this is representative of the study population and perform a simulation study. Fourth, despite the assumption that the lower quarter of the sternum, as a ‘point’, was compressed, the liver was not observed via CT, which was consistent with the results of previous studies [9]. However, in clinical scenario, the chest compression site is not a ‘point’ but rather an ‘area’, which is equivalent to the area of the palm of the hand during manual CPR or the area of the compressing device during mechanical CPR. When the cases were further reviewed for concept, the compressed area from the lower quarter of the sternum to the xyphoid process and the liver were observed on the CT images of 25 patients (43.1%). While our proposed site may increase the compressed single ventricle area by more than 7%, liver injury may be unavoidable in less than half of the patients. Unlike liver damage, which that was predictable, because this was a retrospective study, we could not investigate the incidence of rib fracture or lung injury caused by compression at the right deviation position. Therefore, for these patients, if chest compression for CPR is performed at the location recommended in our study, further studies are needed to evaluate the hemodynamic benefits and the risks for additional injuries, including rib fracture, lung injury, and liver injury. Finally, there may be minor measurement errors.

Because the anterior chest is not a completely flat structure, it may not be possible to provide adequate compression to the ideal compression site using the current conventional compression device (e.g., LUCAS). Therefore, we propose placing an aiding tool to between the conventional compression device and the patient’s chest or between the rescuer’s hands and the patient’s chest. By using an assistive device, it will be possible to transmit the force to the right of the site suggested in our study without significantly deviating from the existing compression site (Fig. S1). We also suggest that further research of the practicality, safety, and efficacy of our proposed compression aids is needed.

## Conclusion

When performing CPR on a patient with Fontan circulation with dextrocardia, right-sided chest compression may be better than at the conventional location.

### Electronic supplementary material

Below is the link to the electronic supplementary material.

Supplementary Material 1

## Data Availability

Correspondence and requests for materials should be addressed to JYJ.
