# Supplementary material for: Ideal chest compression site for cardiopulmonary resuscitation in fontan circulation patients with dextrocardia
Source: BMC Cardiovasc Disord. 2024 Jan 3;24:22. doi: 10.1186/s12872-023-03691-0 (PMC10765782; doi:10.1186/s12872-023-03691-0)

Figure S1. An assist device for transferring force to the right of conventional compression location

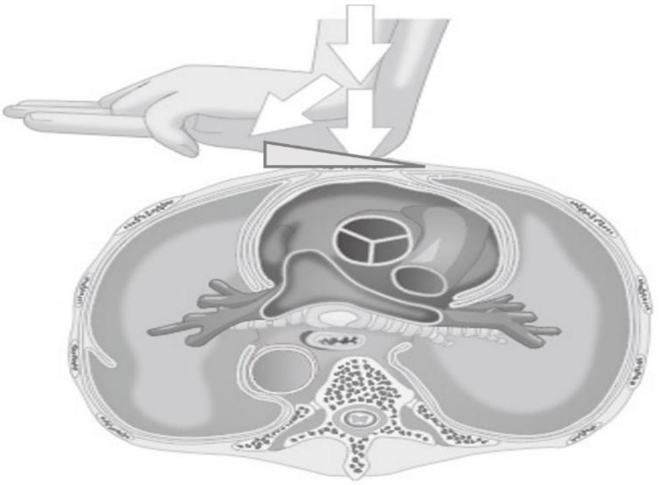

Supplement: Supplementary file 1 — Supplementary Material 1 [file 12872_2023_3691_MOESM1_ESM.pdf]
